# Association of socioeconomic status with hypertension prevalence and control in Nanjing: a cross-sectional study

**DOI:** 10.1186/s12889-022-12799-5

**Published:** 2022-03-02

**Authors:** Zhenzhen Qin, Chao Li, Shengxiang Qi, Hairong Zhou, Jie Wu, Weiwei Wang, Qing Ye, Huafeng Yang, Chenchen Wang, Xin Hong

**Affiliations:** 1 Department of Non-Communicable Disease Prevention, Nanjing Municipal Center for Disease Control & Prevention, 2, zizhulin, Nanjing, 210003 China; 2 Department of Epidemiology, School of Public Health, Nanjing Medical University, Nanjing, 211116 China

**Keywords:** Socioeconomic Status, Hypertension Prevalence, Hypertension Control, Adults, Cross-sectional Study

## Abstract

**Background:**

The role of socioeconomic status (SES) on hypertension prevalence and hypertension control has gotten much attention but with conflicting results. This paper aimed to quantify the association of SES with both hypertension prevalence and hypertension control rate in Nanjing, China.

**Methods:**

A community-based cross-sectional study was conducted using multistage random sampling on 60,283 adults aged more than 18 years between March 2017 and June 2018. Hypertension was defined as systolic blood pressure (BP) ≥ 140 mmHg and/or diastolic BP ≥ 90 mmHg or self-reported diagnosis of hypertension or respondent's report of taking antihypertensive medications. The controlled hypertension was defined by systolic BP < 140 mmHg and diastolic BP of < 90 mmHg among the subjects that self-reported exhibiting hypertensive and taking antihypertensive medications. The associations between SES with hypertension prevalence and hypertension control were quantified using generalized mixed model regression analysis and reported as odds ratios (ORs) and 95% confidence interval (CI).

**Results:**

There was a high prevalence of subjects with primary educational level (49.6%) or unemployed and retired (49.5%) or lower annual household income level (44.9%) in each SES group, respectively. After adjustments for potential confounding factors, there were higher odds of hypertension among those with primary educational level (OR = 1.56), but lower odds for controlled BP (OR = 0.51). Higher odds of hypertension could be found among unemployed and retired, and higher odds of controlled BP was observed in the mental laborers or students (OR = 1.30), compared with the other categories, respectively. The lower-income group was more likely to be hypertensive (OR = 1.35) and less likely to have controlled hypertension (OR = 0.73).

**Conclusion:**

Socioeconomic status played an important role in hypertension prevalence and hypertension control among adults in Nanjing, China. Strategies for hypertension prevention and control should especially focus on people in the vulnerable lower SES groups.

## Background

It is widely considered that high blood pressure (BP) is a major risk factor for heart disease, and it also plays a critical role in the development of stroke, myocardial infarction, heart failure, and renal failure [1]. The lack of awareness and control of hypertension is not unique to China. Globally, hypertension prevalence is markedly witnessed a gradual increase [2]. During the past decades, with the demographic transformation and lifestyle change, China's economy and society have experienced rapid improvement, which has had a huge impact on the health of the population [3]. In recent years, the number of people exposed to metabolic and behavioral risk factors has perceptibly increased [4]. The latest report on cardiovascular diseases (CVDs) in China issued that 25.2% of Chinese adults (approximately 270 million) suffered from hypertension, but the patients had little awareness on hypertension-related knowledge, for example, the diagnostic criteria, the treatment rate, the control rate, risk behaviors and so on [5]. It follows then that hypertension has been a substantial public health problem in China.

The cause of hypertension remains largely unknown, and it is thought to involve genetic, old age, males, obesity, unhealthy lifestyle such as insufficient physical activity (PA), excess sodium intake, and several environmental factors, including the more dramatic socioeconomic status (SES) [1, 6]. Extensive researches have been widely conducted on SES inequalities for the prevention and control of hypertension, and some of the results are controversial [1, 6–13]. More than that, a large number of studies concerning the influence of a single factor of SES on hypertension, reported differences in prevalence and control of hypertension by different dimensions of SES [10–15]. Much more researches are strongly needed to identify the influence of multiple markers of SES (educational attainment, occupational status, and annual household income) on the prevention and control of hypertension.

In this context, we evaluated the association between the three indicators of SES and hypertension prevalence and hypertension control and operating across gender levels in a large number of samples of adults aged over 18 years in Nanjing, China.

## Methods

### Study design and sample approach

A community-based cross-sectional survey was conducted in Nanjing, China between March 2017 and June 2018. In this study, a multistage sampling approach was used to select participants. Firstly, five from twelve districts were randomly selected. Secondly, four administrative streets were randomly chosen through the method of probability proportional to size (PPS) from each selected district. Thirdly, three communities were randomly chosen using PPS from each selected administrative street. Then, one residential group with at least fifty households was chosen from each selected community. Lastly, one person was selected through the application of Kish Grid Sampling from each household. Permanent residents staying more than 6 months aged over 18 years were eligible to participate, excluding pregnant women and those with mental disorders. According to the age composition ratio of the data from the Sixth National Population Census of Nanjing in 2010, the sample numbers of different genders and age groups were calculated to ensure the feasibility of the study. Ultimately, a total of 60,283 adults were recruited to our study.

The study was approved by the Ethics Committee of Nanjing Center for Disease Control and Prevention. Each participant received an information leaflet about the study and signed informed consent. An on-site physician would present and explain the study to all participants.

### Measurements

The investigation content includes a questionnaire survey, body measurement, and laboratory testing. The on-site survey phase is an effort involving live, face-to-face interviews and anthropometric measurements, together with the administration of a questionnaire by trained interviewers. The standard questionnaire, which was designed based on the questionnaire of the China Adult Chronic Disease and Nutrition Surveillance (CACDNS) that was carried out every three years in China [16], contains information about the basic personal information (age, gender, marital status, education level, occupation type, family income, etc.), the prevalence of behavioral risk factors (smoking, alcohol consumption, diet, physical activity), family history of hypertension, etc.

Anthropometric measurements include weight, height, and blood pressure. Bodyweight and height were measured in the fasting state in the early morning. Face-to-face measurement procedures were based on standard protocols. All measurements were conducted by the trained investigators. A standardized scale (RGZ-120/160, I Wish Corporation, China) was used to measure body weight and height to the nearest 0.1 kg and 0.1 cm, respectively, with the height and weight scale placed on a horizontal hard-floor surface. Weight was measured with subjects in light clothing and height was measured with subjects barefooted and his/her back facing toward the pole of the height scale, simultaneously. Then, body mass index(BMI) was calculated by dividing weight in kilograms by height in meters squared(BMI = kg/m^2^). Sitting BP was measured three times by investigators using an electronic sphygmomanometer (HBP-1300, Omron Corporation, Japan) to the nearest 1 mm of mercury (mmHg), with at least 1-min of rest sitting on a chair with one arm supported at the right atrial level and with the other arm placed on the arm support of the chair. All participants were suggested to avoid strenuous exercise, feeding, caffeine, and smoking within 30 min and were seated at least 5 min quietly before BP measurement. The mean BP level of the latter two measurements was calculated and used for the analysis.

The 5 ml fasting venous blood sample was obtained from each participant to estimate the parameter of the serum lipid metabolism including serum levels of triglycerides, total cholesterol, high-density lipoprotein cholesterol, and low-density lipoprotein cholesterol, which were detected by a full-automatic biochemical instrument (BS-800, Mindray Corporation, China) in certified medical laboratories.

### Study variables

#### Outcome variable

The outcomes we examined were hypertension prevalence and controlled hypertension. Hypertension was defined as systolic BP ≥ 140 mmHg (millimeters of mercury) and/or diastolic BP ≥ 90 mmHg or self-reported diagnosis of hypertension or respondent's report of taking antihypertensive medications [17]. The controlled hypertension was defined by systolic BP < 140 mmHg and diastolic BP < 90 mmHg among all the subjects that self-reported exhibiting hypertensive and taking antihypertensive medications.

#### Explanatory variable

SES was treated as the independent variable. Socioeconomic status is one of the most widely studied concepts in the social sciences. Several measurements of socioeconomic status have been proposed. However, the most commonly included quantification are family income, educational attainment, and occupational status. In our study, we collected three factors (education attainment, occupational status, and annual household income) as components of our participants' socioeconomic status (SES). The relevant question such as “*What's your highest degree?*” was designed in our questionnaire to collect information by selecting the setting options. In our analysis, all the three indicators were classified as categorical variables: the education attainment was grouped based on the highest educational level of each participant (primary, junior or senior, and college), the occupational status was summarized from 11 categories (*production personnel in agriculture, equipment operators, business and service people, leaders of enterprise units, public institution staffs, professional technical personnel, professional soldiers, other workers, students, the unemployed, people doing housework, the retired, *etc*.*) then classified into manual laborers, service staffs, mental laborers or students, and unemployed and retired people, annual the household income was divided by tertiles range (lower: < 60,000 ¥, middle: ≤ 60,000 < 108,000 ¥, higher: ≥ 108,000 ¥) according to the subjects’ self-reported annual family income.

### Covariates

Massive studies have been made of plentiful demographic characteristics and lifestyle-related exposures that played a crucial part in the development of hypertension [18]. In our study, the selected covariates considered include gender, age, marriage (single, married or living with a partner, separated, divorced or widowed), smoking (current smoking was defined as having smoked 100 cigarettes in one’s lifetime and currently smoking cigarettes) [19], alcohol drinking (defining a drinker as current drinking was defined as alcohol intake more than once per month during the past 12 months) [19], family history of hypertension, salt intake (the cutoff value of salt intake was 6 g/d), medication (defining medication as taking antihypertensive drug for high blood pressure), PA (defining sufficient PA ≥ 600 MET-min/w) using the International Physical Activity Questionnaire (IPAQ) [20], BMI (categorized individuals as underweight: BMI < 18.5 kg/m^2^, normal:18.5 ≤ BMI < 23.9 kg/m^2^, overweight/obese: BMI ≥ 24.0 kg/m^2^) [21] and dyslipidemia (according to Chinese guidelines for the Prevention and treatment of dyslipidemia in adults, setting total cholesterol (TC) > 6.22 mmol/L and/or high-density lipoprotein cholesterol (HDL-C)) < 1.04 mmol/L and/or low-density lipoprotein cholesterol (LDL-C) > 4.14 mmol/L and/or triglyceride (TG) > 2.26 mmol/L, and/or individuals who has been diagnosed and/or as dyslipidemia [22].

### Statistical analysis

Continuous and categorical variables were expressed as mean (SD) and numbers (percentages) where appropriate. The inter-group difference was compared using the t-test and Chi-square test. All the analyses were conducted based on the overall sample and the stratified sample by gender. The associations between SES with hypertension prevalence and hypertension control were quantified using generalized mixed model regression analysis for each SES indicator in one model, separately. Unadjusted odds ratios (ORs) and 95% confidence intervals (CI) were estimated in random-model by using the college educational level, unemployed and retired, and the higher annual household wealth level as the respective reference categories. Adjusted ORs were calculated by adding gender, age, marriage, smoking, alcohol drinking, family history, salt intake, physical activity, BMI, and dyslipidemia as covariates. Then, we conducted a stratified analysis by gender with assessing the ORs in males and females, separately. A value of *P* < 0.05 was considered significant.

The data were double-entered and cleaned with Epi Data 3.1 (The Epi Data Association 2008, Odense, Denmark) and analyzed by SPSS version 20.0 for Windows (SPSS Inc., Chicago, IL, USA).

## Results

The participants’ characteristics were shown in Table 1. 60,283 (43.7 ± 16.4 years) adults aged over 18 years completed the survey by 29,848 males (49.5%) and 30,435 females (50.5%). Females (44.0 ± 16.1 years) were slightly older than males (43.4 ± 16.7 years) and there was a significant difference between gender groups (*P* < 0.001). The mean values of systolic and diastolic blood pressure were higher in males (*P* < 0.001). The prevalence of hypertension was 26.0%, and it was higher in males (28.7%) than in females (23.4%). The hypertension control rate was 61.1%, while was higher in females (63.2%) than in males (59.4%). There were significant differences in the characteristics including marriage, education attainment, occupation status, annual household income, smoking status, alcohol drinking, family history, salt intake, physical activity, BMI, and dyslipidemia between genders (*P* < 0.001).

**Table 1:** Main characteristics of the study participants recruited

Characteristics	Overall	Male	Female	*P* value
	***N *= 60,283**	***N* = 29,848**	***N* = 30,435**	
**age, mean (SD)**	43.7 (16.4)	43.4 (16.7)	44.0 (16.1)	< 0.001
**Systolic blood pressure (mmHg), mean (SD)**	123.5 (18.9)	125.7 (17.4)	121.3 (20.1)	< 0.001
**Diastolic blood pressure (mmHg), mean (SD)**	77.3 (14.0)	78.8 (14.0)	75.9 (13.8)	< 0.001
**Hypertension prevalence, n (%)**	15,686 (26.0)	8579 (28.7)	7107 (23.4)	< 0.001
**Hypertension control rate, n (%)**^a^	5797 (61.1)	3032(59.4)	2765 (63.2)	< 0.001
**Marriage, n (%)**				
Single	10,556 (17.5)	6043 (20.2)	4513 (14.8)	< 0.001
Married or living with a partner	47,536 (78.9)	23,039 (77.2)	24,497 (80.5)	
Separated, divorced or widowed	2191 (3.6)	766 (2.6)	1425 (4.7)	
**Education, n (%)**				
College	27,805 (46.1)	14,626 (49.0)	13,179 (43.3)	< 0.001
Junior/Senior	27,020 (44.8)	13,434 (45.0)	13,586 (44.6)	
Primary	5458 (9.1)	1788 (6.0)	3670 (12.1)	
**Occupation, n (%)**				
Unemployed and retired people	16,516 (27.4)	5841 (19.6)	10,675 (35.1)	< 0.001
Mental laborers/Students	23,241 (38.6)	12,853 (43.1)	10,388 (34.1)	
Service staff	7192 (11.9)	3489 (11.7)	3703 (12.2)	
Manual laborers	13,334 (22.1)	7665 (25.7)	5669 (18.6)	
**Annual household income,tertiles range(¥)**^**b**^				
Higher (≥ 108,000)	4586 (34.3)	2212 (35.7)	2374 (33.2)	< 0.001
Middle (≤ 60,000 < 108,000)	5411 (40.5)	2560 (41.3)	2851 (39.9)	
Lower (< 60,000)	3358 (25.1)	1430 (23.1)	1928 (27.0)	
**Smoking, n (%)**				
Nonsmoker	46,861 (77.7)	16,878 (56.5)	29,983 (98.5)	< 0.001
Smoker	13,422 (22.3)	12,970 (43.5)	452 (1.5)	
**Alcohol drinking, n (%)**				
Non drinker	42,631 (70.7)	15,616 (52.3)	27,015 (88.8)	< 0.001
Drinker	17,652 (29.3)	14,232 (47.7)	3420 (11.2)	
**Family history, n (%)**				
No	32,565 (61.9)	16,312 (63.3)	16,253 (60.6)	< 0.001
Yes	20,011 (38.1)	9462 (36.7)	10,549 (39.4)	
**Salt intake, n (%)**				
Lower (< 6 g)	10,546 (17.5)	5081 (17.0)	5465 (18.0)	0.003
Higher (≥ 6 g)	49,737 (82.5)	24,767 (83.0)	24,767 (83.0)	
**Physical activity, n (%)**				
Sufficient	49,548 (82.2)	24,238 (81.2)	25,310 (83.2)	< 0.001
Insufficient	10,735 (17.8)	5610 (18.8)	5125 (16.8)	
**BMI(kg/m**^**2**^**), n (%)**				
Underweight	2551 (4.2)	786 (2.6)	1765 (5.8)	< 0.001
Normal	31,810 (52.8)	13,811 (46.3)	17,999 (59.1)	
Overweight/obese	25,922 (43.0)	15,251 (51.1)	10,671 (35.1)	
**Dyslipidemia, n (%)**				
No	43,190 (71.6)	20,663 (69.2)	22,527 (74.0)	< 0.001
Yes	17,093 (28.4)	9185 (30.8)	7908 (26.0)	

^a^The controlled hypertension was defined by systolic BP < 140 mmHg and diastolic BP < 90 mmHg among the subjects that self-reported exhibiting hypertensive and taking antihypertensive medications

^b^Annual household income was collected from 13,355 subjects providing verified information about their family's income(male 6202, female 7153)

Table 2 presents the relation between SES and hypertension for overall participants and subpopulations of each gender. For the overall study sample, after adjustment for potential confounding factors and communities-level clustering effects, the odds ratios were 1.56 (95% CI: 1.43,1.70) and 1.41 (95%CI: 1.33,1.49) with subjects receiving primary education or junior/senior education compared with people receiving college education of developing hypertension. Compared to the unemployed and retired population, participants were significantly less likely to suffer from hypertension in the mental laborers and students (OR = 0.70, 95% CI: 0.65,0.75), service staffs (OR = 0.76, 95% CI: 0.70,0.83), and manual laborers (OR = 0.88, 95% CI: 0.83,0.94), respectively. The odds of developing hypertension were 1.35-fold (OR = 1.35, 95% CI: 1.19,1.52) in adults with lower annual household income as compared with those with higher annual household income. There were no significant differences between the middle annual household income group and the higher (OR = 1.10, 95% CI: 0.98,1.22). Stratified analyses suggested similar SES-hypertension associations by gender.

**Table 2:** Association between socioeconomic status and the prevalence of hypertension among adults in Nanjing

Characteristics	Overall (*N* = 60,283)			Male(*N* = 29,848)			Female (*N* = 30,435)		
	**individuals with hypertension (n/%)**	**OR(95%CI)**	**Adjusted OR(95%CI)**^**b**^	**individuals with hypertension (n/%)**	**OR(95%CI)**	**Adjusted OR(95%CI)**^**c**^	**individuals with hypertension (n/%)**	**OR(95%CI)**	**Adjusted OR(95%CI)**^**ǂ**^
**Education, n (%)**									
College	3491 (12.6)	Ref	Ref	2487 (17.0)	Ref	Ref	1004 (7.6)	Ref	Ref
Junior/Senior	9486 (35.1)	3.94 (3.77,4.12)	1.41 (1.33,1.49)	5161 (38.4)	3.15 (2.98,3.33)	1.28 (1.19,1.38)	4325 (31.8)	6.04 (5.60,6.51)	1.62 (1.48,1.78)
Primary	2709 (49.6)	7.19 (6.74,7.67)	1.56 (1.43,1.70)	931 (52.1)	5.55 (5.01,6.16)	1.40 (1.23,1.59)	1778 (48.4)	12.17 (11.08,13.36)	1.75 (1.55,1.97)
**Occupation, n (%)**									
Unemployed and retired people	8172 (49.5)	Ref	Ref	3493 (59.8)	Ref	Ref	4679 (43.8)	Ref	Ref
Mental laborers/Students	3129 (13.5)	0.14 (0.15,0.16)	0.70 (0.65,0.75)	2275 (17.7)	0.14 (0.13,0.15)	0.74 (0.67,0.81)	854 (8.2)	0.11 (0.10,0.12)	0.64 (0.58,0.71)
Service staff	1113 (15.5)	0.19 (0.17,0.20)	0.76 (0.70,0.83)	639 (18.3)	0.15 (0.14,0.17)	0.73 (0.64,0.83)	474 (12.8)	0.18 (0.17,0.21)	0.83 (0.73,0.94)
Manual laborers	3272 (24.5)	0.33 (0.31,0.35)	0.88 (0.83,0.94)	2172 (28.3)	0.26 (0.25,0.28)	0.89 (0.81,0.98)	1100 (19.4)	0.31 (0.28,0.33)	0.88 (0.81,0.97)
**Annual household income,tertiles range(¥)**^a^									
Higher (≥ 108,000)	1242 (27.1)	Ref	Ref	731 (33.0)	Ref	Ref	511 (21.5)	Ref	Ref
Middle (≤ 60,000 < 108,000)	2139 (39.5)	1.81 (1.65,1.97)	1.10 (0.98,1.22)	1129 (44.1)	1.65 (1.46,1.87)	1.02 (0.88,1.19)	1010 (35.4)	2.03 (1.79,2.31)	1.16 (0.99,1.36)
Lower (< 60,000)	1508 (44.9)	2.23 (2.02,2.46)	1.35 (1.19,1.52)	715 (50.0)	2.05 (1.78,2.36)	1.41 (1.18,1.68)	793 (41.1)	2.58 (2.25,2.97)	1.29 (1.08,1.53)

^a^Annual household income was collected from 13,355 subjects providing verified information about their family's income(male 6202,female 7153)

^b^Mixed model analysis with adjusted for age, gender, marriage, smoking, alcohol drinking, family history, salt intake, physical activity, BMI and dyslipidemia

^c^Mixed model analysis with adjusted for age, marriage, smoking, alcohol drinking, family history, salt intake, physical activity, BMI and dyslipidemia

We further investigated the association between socioeconomic status classification and controlled hypertension (Table 3). After adjustments for potential confounding factors, the odds of controlled hypertension were 0.76 times (0.67 to 0.86) for subjects with Junior/Senior school education than subjects with college school education, and subjects with primary school education showed 0.51 times (0.44 to 0.59), respectively. The odds of controlled hypertension were higher in the mental laborers /students category (OR = 1.30, 95% CI: 1.13,1.48) and lower in the manual laborers’ category (OR = 0.75, 95% CI: 0.67,0.85), compared with the unemployed and the retired, respectively. The odds of controlled hypertension decreased 0.27-fold (OR = 0.73, 95% CI: 0.59,0.90) in adults with lower annual household income as compared with those with higher annual household income. Further stratification by gender, the association of all the SES categories and controlled hypertension remained relatively stable except the lower annual household income category in females.

**Table 3:** Association between socioeconomic status and blood pressure control in reported exhibiting hypertensive patients^a^

Characteristics	Overall (*N* = 9485)			Male(*N* = 5108)			Female (*N* = 4377)		
	**individuals with hypertension control(n/%)**	**OR(95%CI)**	**Adjusted OR(95%CI)**^c^	**individuals with hypertension control(n/%)**	**OR(95%CI)**	**Adjusted OR(95%CI)**^d^	**individuals with hypertension control(n/%)**	**OR(95%CI)**	**Adjusted OR(95%CI)**^**ǂ**^
**Education, n (%)**									
College	1205 (66.9)	Ref	Ref	849 (65.0)	Ref	Ref	356 (72.1)	Ref	Ref
Junior/Senior	3690 (62.0)	0.78 (0.69,0.87)	0.76 (0.67,0.86)	1885 (58.9)	0.74 (0.65,0.86)	0.76 (0.66,0.88)	1805 (65.7)	0.73 (0.59,0.91)	0.80 (0.64,0.99)
Primary	902 (52.0)	0.53 (0.46,0.61)	0.51 (0.44,0.59)	298 (49.7)	0.52 (0.43,0.64)	0.54 (0.43,0.66)	604 (53.2)	0.45 (0.35,0.57)	0.51 (0.39,0.65)
**Occupation, n (%)**									
Unemployed and retired people	3488 (60.9)	Ref	Ref	1512 (58.7)	Ref	Ref	1976 (62.7)	Ref	Ref
Mental laborers/Students	1089 (67.6)	1.35 (1.19,1.52)	1.30 (1.13,1.48)	758 (65.2)	1.32 (1.14,1.53)	1.24 (1.04,1.48)	331 (74.0)	1.68 (1.34,2.11)	1.45 (1.14,1.85)
Service staff	289 (62.0)	1.05 (0.87,1.28)	0.99 (0.81,1.22)	158 (57.9)	1.00 (0.77,1.29)	0.94 (0.72,1.24)	131 (67.9)	1.22 (0.89,1.67)	1.07 (0.77,1.48)
Manual laborers	931 (55.4)	0.78 (0.69,0.87)	0.75 (0.67,0.85)	604 (55.2)	0.84 (0.72,0.97)	0.79 (0.68,0.94)	327 (55.7)	0.74 (0.61,0.88)	0.68 (0.56,0.82)
**Annual household income,tertiles range(¥)**^b^									
Higher (≥ 108,000)	449 (59.2)	Ref	Ref	259 (58.2)	Ref	Ref	190 (60.5)	Ref	Ref
Middle (≤ 60,000 < 108,000)	931 (58.3)	0.96 (0.80,1.16)	0.99 (0.82,1.20)	434 (57.6)	0.97 (0.76,1.24)	0.98 (0.77,1.26)	397 (59.2)	0.95 (0.71,1.26)	1.02 (0.76,1.37)
Lower (< 60,000)	471 (50.2)	0.72 (0.59,0.88)	0.73 (0.59,0.90)	205 (46.4)	0.63 (0.48,0.82)	0.63 (0.48,0.84)	266 (53.6)	0.79 (0.59,1.07)	0.82 (0.59,1.12)

^a^The controlled hypertension was defined by systolic BP < 140 mmHg and diastolic BP < 90 mmHg among the subjects that self-reported exhibiting hypertensive and taking antihypertensive medications

^b^Annual household income was collected from 3122 subjects providing verified information about their family's income(male1641,female 1481)

^c^Mixed model analysis with adjusted for age, gender, marriage, smoking, alcohol drinking, family history, salt intake, physical activity, BMI and dyslipidemia

^d^Mixed model analysis with adjusted for age, marriage, smoking, alcohol drinking, family history, salt intake, physical activity, BMI and dyslipidemia

## Discussion

This study contributes to current researches investigating the association between SES with both hypertension prevalence and hypertension control rate. We found roughly steep gradients of the association between educational attainment, occupational status, and annual household income with hypertension prevalence and hypertension control rate.

Delayed diagnosis inevitably leads to delayed treatment and control, which can have serious health consequences. As is well-known, the harmful effects of high blood pressure can be contained if blood pressure is under control. For example, the mortality of cardiovascular or cerebrovascular diseases can be decreased by hypertension control [23]. However, hypertension is more difficult to control as an increased chronic disease than acute infectious diseases in current China during the epidemiological transition.

In recent years, the medical system reform was further deepened, and more attention were paid to the health inequality in China. Nowadays, both the prevalence and the treatment of hypertension are related to the imbalance of social and economic levels. In addition to individual factors, the comprehensive level of regional development also has an important impact on people’s health. The allocation and accessibility of medical and health resources, as well as different influencing factors such as the social insurance system, could affect the occurrence and development of chronic diseases in China. However, there is a lack of effective research with a large scale of data focusing on the association of individual factors affecting by regional economic development and hypertension in terms of prevalence and the control, especially the regulation determinants of individual SES in Nanjing, China. Understanding the factors related to SES is important for guiding the implementation of hypertension prevention and control policies.

Evidence showed the low-SES population was known to have an unhealthy lifestyle resulting in the development of poor eating habits, influencing behaviors related to cigarette smoking and alcohol consumption, as well as insufficient PA [24]. As a final result, they were more likely to be exposed to numerous risk factors and, therefore, causing an obviously excessive burden of disease including hypertension, while the three components of SES might explain the causes of the consequence [25]. Education levels are determined in early adulthood and generally remain constant, unlike the other socioeconomic indicators that are more easily changed. For this reason, education is most widely used in the measurement of SES in epidemiological studies [26]. In our analysis, educational attainment was an important SES indicator, which was observed to have a strong association with the prevalence and control rate of hypertension. The result was consistent with the previous studies [27, 28]. People with higher education may have more chances to know health knowledge on hypertension and subsequently have a healthier lifestyle, like they were more likely to take exercise, to drink moderately, to receive preventive medical care, and were less likely to smoke [29], to keep blood pressure steady, which could decrease or delay the occurrence of hypertension or the complications of hypertension [23, 30, 31]. Education structures occupation type and income to a certain extent. Thus, to control the incidence of hypertension in adults, one major step is to increase their awareness of health care, especially behavior intervention in the low education population. Likewise, more strategies should be enhanced on promoting the level of education universally and urgently. Compared to the manual laborers, subjects who worked as service staff or mental laborers/students had a relatively lower prevalence rate of hypertension in our study, while the elderly retired people and the unemployed reported a reverse higher risk of getting hypertension. A plausible interpretation is that the hypertensive factors, such as social psychological factors, working overtime, high temperature and noisy environment, and other occupational factors are more common in manual laborers [32, 33]. By using the advantages of occupational sites, implementing interventions for high-risk groups and the whole population to reduce cardiovascular risk factors is possible to achieve the greater effect of preventing cardiovascular diseases as well as reducing the burden of cardiovascular diseases. From this, it is undoubtedly an effective way to carry on some health related lectures as well as early screening of hypertension among the occupational population to raise awareness to control and prevent hypertension. Higher annual household income indicated decreasing risk of hypertension and an increasing control rate in our study, which was in line with the existing research results [34]. This could be attributed to the fact that people with higher income can contact with precise healthcare systems more frequently. Furthermore, they may be more interested in maintaining a good body through proper exercise, a balanced diet, and a healthy lifestyle. In other words, people with higher annual household incomes are more likely to live or work in a healthy environment thus have a greater chance of a delayed onset of developing high blood pressure or having their blood pressure controlled adequately.

Regarding the strengths of this study, this survey included a large number of representative subjects in Nanjing, China.The second strength is that, in addition to gender, age, marriage, smoking, alcohol drinking, family history, salt intake, physical activity, BMI, and dyslipidemia, community-level potential clustering-effects were also considered in using the mixed-effects regression analysis.

Several limitations of our study must be noted when interpreting these findings. First, the association between SES and hypertension does not imply any causal relationship, as it was observed in a cross-sectional study. The second might be the potential recall bias because the hypertension information at the time of filling out the questionnaire was self-reported by the subjects. Thirdly, there might be other factors that were not considered in our analysis. Therefore, reliable cohort studies are needed to verify our results in the future.

In conclusion, socioeconomic status played an important role in hypertension prevalence and hypertension control among adults in Nanjing, China. Strategies for hypertension prevention and control should especially focus on people in the vulnerable lower SES groups.

## Data Availability

Availability of data and materials may be made available upon reasonable request from the corresponding author.

## Abbreviations

SES: Socioeconomic status
BP: Blood pressure;OR:Odds ratio
CI: Confidence interval
CVDs: Cardiovascular diseases
PA: Physical activity
PPS: Probability proportion to size
BMI: Body mass index
TC: Total cholesterol
HDL-C: High-density lipoprotein cholesterol
LDL-C: Low-density lipoprotein cholesterol
TG: Triglyceride
